# The Usefulness of the Glucose Management Indicator in Evaluating the Quality of Glycemic Control in Patients with Type 1 Diabetes Using Continuous Glucose Monitoring Sensors: A Cross-Sectional, Multicenter Study

**DOI:** 10.3390/bios15030190

**Published:** 2025-03-16

**Authors:** Sandra Lazar, Ovidiu Potre, Ioana Ionita, Delia-Viola Reurean-Pintilei, Romulus Timar, Andreea Herascu, Vlad Florian Avram, Bogdan Timar

**Affiliations:** 1 First Department of Internal Medicine, “Victor Babes” University of Medicine and Pharmacy, 300041 Timisoara, Romania; sandra.lazar@umft.ro (S.L.); ionita.ioana@umft.ro (I.I.); 2 Department of Hematology, Emergency Municipal Hospital, 300254 Timisoara, Romania; 3 Centre for Molecular Research in Nephrology and Vascular Disease, “Victor Babes” University of Medicine and Pharmacy, 300041 Timisoara, Romania; timar.romulus@umft.ro (R.T.); andreea.herascu@umft.ro (A.H.); avram.vlad@umft.ro (V.F.A.); bogdan.timar@umft.ro (B.T.); 4 Multidisciplinary Research Center for Malignant Hematological Diseases (CCMHM), Victor Babes University of Medicine and Pharmacy, 300041 Timisoara, Romania; 5 Department of Medical-Surgical and Complementary Sciences, Faculty of Medicine and Biological Sciences, “Stefan cel Mare” University, 720229 Suceava, Romania; delia.pintilei@usm.ro; 6 Department of Diabetes, Nutrition and Metabolic Diseases, Consultmed Medical Centre, 700544 Iasi, Romania; 7 Second Department of Internal Medicine, “Victor Babes” University of Medicine and Pharmacy, 300041 Timisoara, Romania; 8 Department of Diabetes, “Pius Brinzeu” Emergency Hospital, 300723 Timisoara, Romania

**Keywords:** glucose management indicator, HbA1c, biomarker, continuous glucose monitoring system, type 1 diabetes mellitus

## Abstract

The Glucose Management Indicator (GMI) is a biomarker of glycemic control which estimates hemoglobin A1c (HbA1c) based on the average glycemia recorded by continuous glucose monitoring sensors (CGMS). The GMI provides an immediate overview of the patient’s glycemic control, but it might be biased by the patient’s sensor wear adherence or by the sensor’s reading errors. This study aims to evaluate the GMI’s performance in the assessment of glycemic control and to identify the factors leading to erroneous estimates. In this study, 147 patients with type 1 diabetes, users of CGMS, were enrolled. Their GMI was extracted from the sensor’s report and HbA1c measured at certified laboratories. The median GMI value overestimated the HbA1c by 0.1 percentage points (*p* = 0.007). The measurements had good reliability, demonstrated by a Cronbach’s alpha index of 0.74, an inter-item correlation coefficient of 0.683 and an inter-item covariance between HbA1c and GMI of 0.813. The HbA1c and the difference between GMI and HbA1c were reversely associated (Spearman’s r = −0.707; *p* < 0.001). The GMI is a reliable tool in evaluating glycemic control in patients with diabetes. It tends to underestimate the HbA1c in patients with high HbA1c values, while it tends to overestimate the HbA1c in patients with low HbA1c.

## 1. Introduction

Continuous glucose monitoring systems (CGMS) are medical devices that provide real-time glycemic measurements in a continuous manner with a predefined periodicity, typically ranging from five to ten minutes [1]. Taking this into account, these systems offer up to nearly 300 readings per day and utilize the variable electrical conductivity of an electrode placed subcutaneously, which is dependent on the patient’s interstitial glucose concentrations. Based on the glucose concentration in the interstitial tissue, these devices estimate the patient’s blood sugar levels. There are various available CGMS options, including holter, real time and flash type, most of which feature a transmitter that is attached to the electrode that relays data to a receiver, such as an insulin pump, mobile phone or another external device [2,3,4].

Despite being designed to measure glycemic values from interstitial fluid, CGMS can deliver estimations of blood glucose (BG) with adequate accuracy for daily use, if they are employed in accordance with the manufacturer’s guidelines for calibrations, sensor replacement or potential treatment interactions [5,6].

In comparison to using self-monitoring blood glucose (SMBG) alone, using CGMS necessitates fewer daily finger pricks for glucose measurement. By providing real-time information about the patient’s blood sugar, as well as alerts for hyperglycemic or hypoglycemic events, and offering insights regarding glycemia’s evolutionary trend, CGMS contribute significantly to achieving improved glycemic control [7,8].

The use of CGMS has proven to be valuable in both type 1 (T1DM) and type 2 diabetes mellitus (T2DM) [7,8]. However, their importance is emphasized in patients with T1DM because the fluctuations in glycemic levels are considerably more pronounced in these patients. This is primarily due to factors that influence glycemia, such as hormonal changes, diet, exercise and infections, in addition to the pathogenesis of T1DM, which is characterized by a complete absence of endogenous insulin and thus needs precise exogenous replacement. In this case, even a slightly higher dose of insulin than needed may lead to the development of hypoglycemia, while a slightly lower than needed insulin dose leads to increases in BG levels. This results in a more significant increase in glycemic fluctuations compared to T2DM, where although insulin resistance is increased, there is still a residual pancreatic insulin secretion which acts like a buffer, minimizing the glycemic variability (GV) phenomenon [9,10,11].

The widespread use of CGMS in contemporary medical practice has facilitated the introduction of the concept of GV, which pertains to fluctuations in parameters connected to glucose homeostasis over a specific period. Recent studies have revealed a significant association between severe glycemic oscillations and the risk of developing long-term complications associated with diabetes mellitus (DM) [12,13,14]. As a result, diabetes management in recent years has focused not only on achieving an optimal average glycemic value, specifically an optimal HbA1c level, but also on maintaining stable glycemic oscillations [15,16].

Along with these indicators, current CGMS provide a surrogate indicator derived from recorded glycemic data over a long period of time (minimum over 10 days), through which an inferential estimate of HbA1c laboratory measurement can be made. For most CGMS, this estimate is called the Glucose Management Indicator (GMI). The GMI provides a quick overview of a patient’s glycemic control in a timely manner, without the need for blood sample collection or laboratory tests. On the other hand, the GMI provides only an approximate, inferential value, derived from the CGMS glycemic measurement. Therefore, it depends on the accuracy of the CGMS and on the wear time adherence in the analyzed period [17,18,19].

The HbA1c values depend on the lifespan of erythrocytes and are influenced by various pathologies such as hemoglobinopathies, while GMI is independent of erythrocyte turnover. At the same time, HbA1c measurement requires a visit to a laboratory and the drawing of a blood sample, whereas GMI is readily available in every CGMS report for a patient who has worn the sensor for an adequate period [20,21].

On the other hand, interruptions in the use of the CGM system are common due to intrinsic or extrinsic factors. Additionally, since the GMI indirectly evaluates BG levels by measuring interstitial glucose concentration, deviations from the real average glycemic values may occur when using the GMI [22,23].

The GMI value is expressed as a percentage and is generally determined by applying the formula GMI (%) = 3.31 + 0.02392 × mean glucose in mg/dL. This equation was established through the regression analysis between HbA1c values and mean sensor glycemic readings [24].

The aim of this study was to evaluate to what extent the GMI reflects the real HbA1c, as measured by standardized laboratory assay, what is the degree of error of the estimate and what factors are associated with a higher probability of GMI estimation error among patients with T1DM. Also, this study aimed to evaluate the relationship between GMI and HbA1c by evaluating the correlation, concordance and internal consistency between the two variables, as well as to identify possible discrepancies between the two indicators.

## 2. Materials and Methods

### 2.1. Study Design and Patients

In a cross-sectional study design, 147 patients with T1DM were enrolled, according to a consecutive case, population-based study design, in two diabetes clinics in Romania: The Diabetes Centre of the “Pius Brinzeu” Emergency Hospital from Timisoara, Romania (105 patients enrolled), and Consultmed Clinic, Iasi, Romania (42 patients enrolled). All patients were previously treated with insulin for at least one year prior to enrollment in this study and the insulin’s route of administration was unchanged during the 6 months prior to enrollment: 57.1% (84 patients) used basal/bolus insulin delivery regimens with multiple daily insulin injections, combining a basal insulin analogue (Tresiba, Toujeo or Lantus insulins) with prandial rapid or second-generation rapid insulin analogues (Fiasp, Lyumjev, Novorapid or Humalog insulins). The participants had a median age of 32 years (interquartile distance 15 years), a median HbA1c of 7.0% (interquartile distance 0.9 percentage points) and gender distribution was 40.8% (60) men and 59.2% (87 women). The median body mass index in the cohort was 22.6 kg/m^2^ (interquartile distance 6.2 kg/m^2^).

This study was conducted in accordance with the Declaration of Helsinki and approved by the independent Ethics Committee of the “VICTOR BABES” UNIVERSITY OF MEDICINE AND PHARMACY, Timisoara, Romania (Approval Number 50 from 19 October 2021). Informed consent was obtained from all subjects involved in this study.

### 2.2. Clinical, Laboratory and Anthropometric Measurements

In all participants, data regarding their age, diabetes duration, type of insulin regimen used and gender were collected from their medical record. HbA1c was measured on the day of the visit, using the chemiluminescence DCCT-standardized method. During the patient’s visit, anthropometric data were collected: body weight, height and body mass index.

### 2.3. Continuous Glucose Monitoring

All patients wore a CGM system for at least 80% of the time in the last 90 days. The reports, which included the GMI, were generated using the data collected in the last 90 days. The CGMS measurements were performed using either a Medtronic Guardian 3 (for standalone CGMS users) or a Medtronic Guardian Link (for sensor-augmented insulin pump users), systems which used the same type of glycemic sensor, provided by Medtronic (sensor product code MMT-7020C5).

### 2.4. Statistical Analysis

Data were collected and analyzed using MedCalc for Windows statistical software, version 19.4 (MedCalc Software, Ostend, Belgium) and Statistical Package for Social Sciences (SPSS) version 29 (IBM Corporation, Armonk, New York, NY, USA). The results are presented as median values and corresponding interquartile distance (continuous variables with non-parametric distribution) or absolute frequencies and percentage from the subgroup’s total (nominal variables). The distribution of the continuous variables was evaluated using the Shapiro–Wilk method.

To evaluate the significance of the differences between medians of continuous variables, the related-samples Wilcoxon Signed Rank test (paired variables) was used. The confidence interval of the differences (95%) for continuous, non-parametrically distributed variables was calculated using the Hodges–Lehmann related-samples method. The concordance between measurements was evaluated using the Kendall’s W coefficient of concordance and its corresponding statistical significance.

The reliability of GMI use instead of HbA1c was evaluated using the Cronbach’s alpha value between the two separated methods of measurement, the intraclass correlation coefficients, and Friedman’s and Tukey’s tests for nonadditivity, while the inter-agreement between measurements was analyzed by calculating the Cohen’s weighted kappa using quadratic weights. To evaluate the relationship between continuous variables, regression models were built, their strengths being assessed using the Spearman’s correlation coefficient.

The sample size was previously calculated to provide a statistical power for the main outcome of at least 80% (1 − β = 0.80) in parallel with a confidence level of 95% (1 − α = 0.95). According to these parameters, for an anticipated median difference between GMI and HbA1c of 0.2 with an interquartile distance of 0.15 percentage points, a sample size of 131 patients resulted. According to these inferential assumptions, in this study, a *p*-value lower than 0.05 was considered the threshold for statistical significance.

## 3. Results

**Figure 1:**
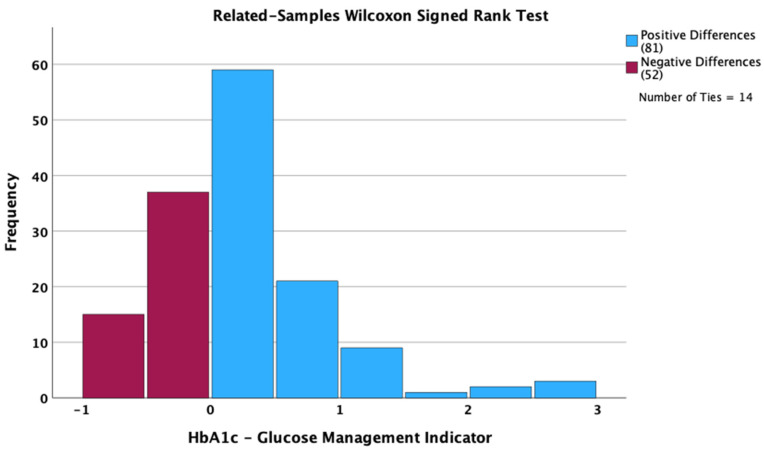
Differences between HbA1c and Glucose Management Indicator.

The median GMI value was higher by 0.1 percentage points than the median HbA1c value (7.0 vs. 6.9 percentage points). Despite not being clinically significant, the differences between the two medians were statistically significant (*p* = 0.007; related-samples Wilcoxon Signed Rank test). The values for both GMI and HbA1c had an interquartile distance of 0.9 percentage points. Based on the related-samples Hodges–Lehmann method, the estimated median difference 95% confidence interval was between 0.05 and 0.2 percentage points; this interval did not include the zero value and led to a significant difference. Regarding the individual paired differences between the HbA1c and GMI values, we observed 14 ties, 81 positive differences and 52 negative differences (Figure 1).

**Figure 2:**
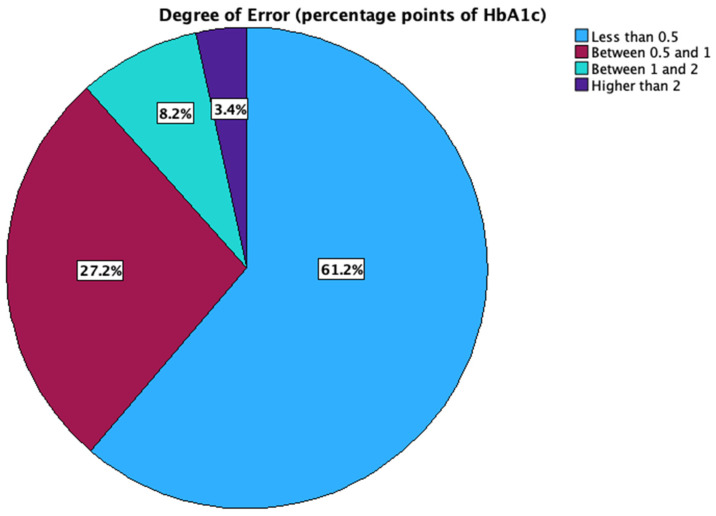
Distribution of the magnitude of GMI error.

In the studied group, 61.2% of the patients had a difference between HbA1c and GMI of less than 0.5 HbA1c percentage points, 27.2% between 0.5 and 1 HbA1c percentage points, 8.2% between 1 and 2 percentage points, and 3.4% higher than 2 HbA1c percentage points (Figure 2).

**Figure 3:**
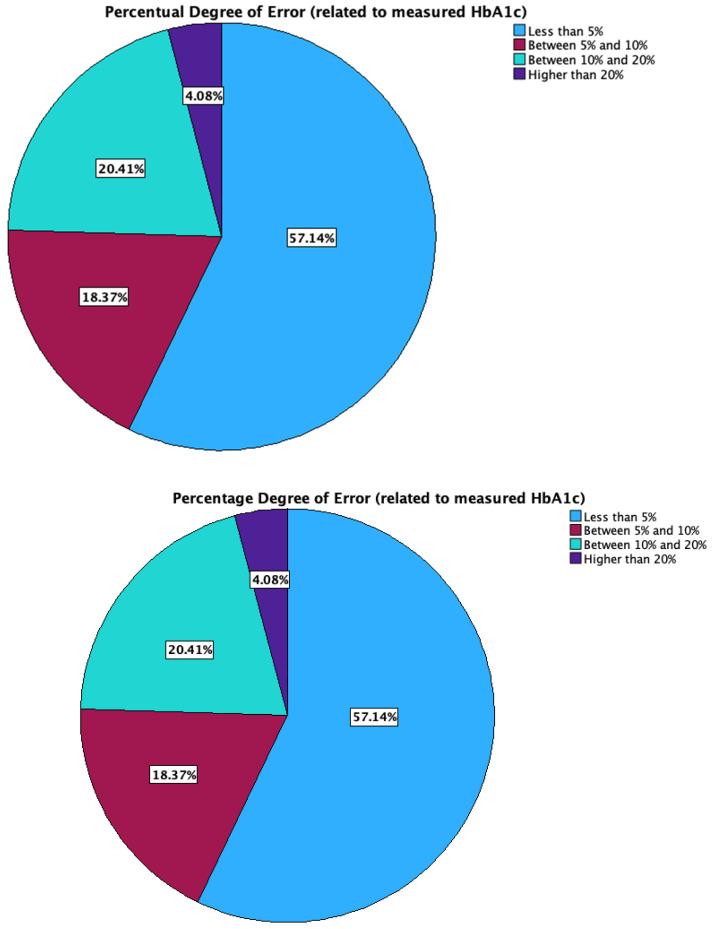
Distribution of the percentage GMI estimation error.

Regarding the percentage differences obtained for GMI versus the measured HbA1c, 57.1% of the patients had a GMI which differed from HbA1c by less than 5%, 18.4% between 5% and 10%, 20.4% between 10% and 20%, and 4.1% higher than 20% (Figure 3).

**Figure 4:**
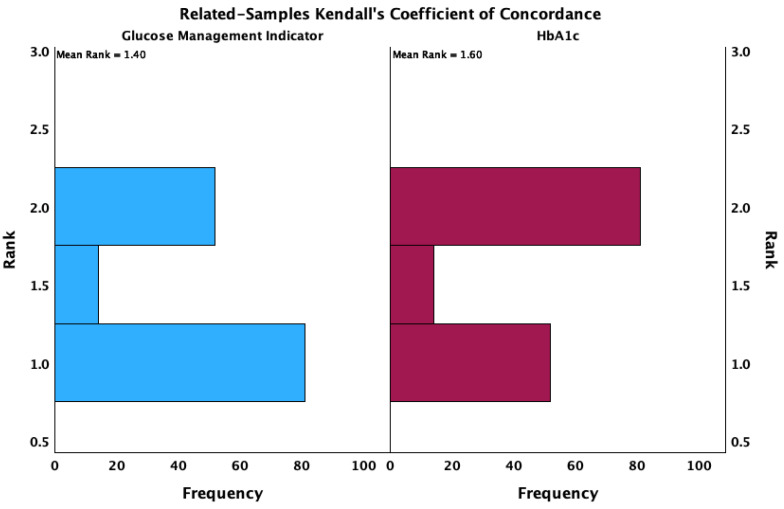
Concordance analysis between GMI and HbA1c.

The obtained Kendall’s W coefficient of concordance was 0.043 (*p* = 0.012) with a test statistic of 6.323 (Figure 4).

**Figure 5:**
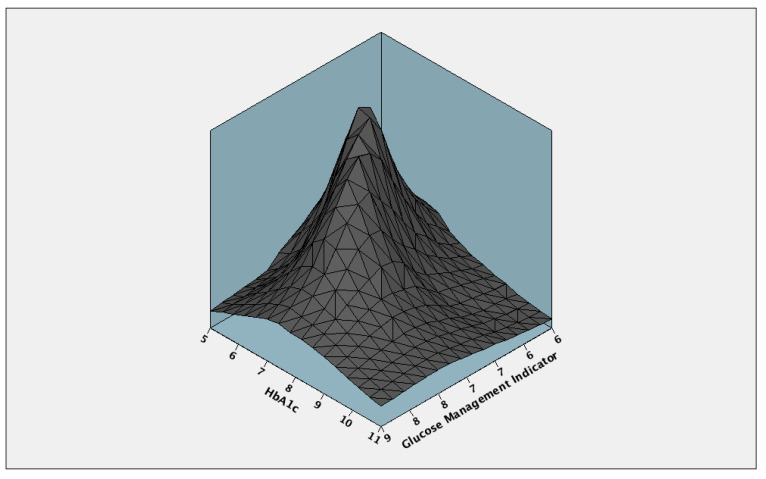
Inter-item covariance between GMI and HbA1c.

The two measurements proved to have a good reliability, demonstrated by a Cronbach’s alpha index of 0.74, with an inter-item correlation coefficient of 0.683 and an inter-item covariance between HbA1c and GMI of 0.813 (Figure 5). Within measurements, the between-items sum of squares obtained using Friedman’s and Tukey’s tests for nonadditivity was 2.178, with a corresponding Friedman’s Chi-Squared of 9.808 (*p* = 0.002). When analyzing the concordance between GMI and HbA1c values, intraclass correlation coefficients for single measurements of 0.587 [0.47–0.68] 95% CI (*p* < 0.001) and for average measurements of 0.74 [0.64–0.81] 95% CI (*p* < 0.001) were obtained. The Cohen’s weighted kappa using quadratic weights for inter-agreement regarding GMI vs. HbA1c measurements was 0.67 [0.59–0.75] 95% CI (*p* < 0.001).

**Figure 6:**
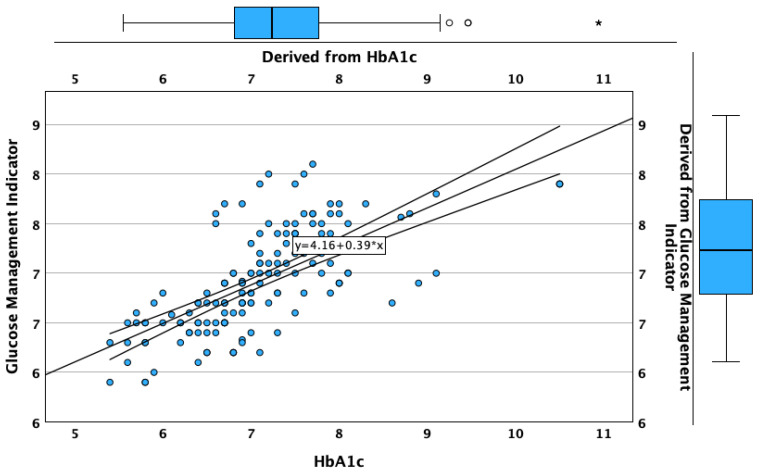
Correlation and regression analysis between HbA1c and GMI.

The regression analysis built using the measured HbA1c value as the independent variable and GMI value as the dependent variable showed that 46.6% of the GMI variation was explained by the variation of the HbA1c, the univariate regression between these two variables being statistically significant (unstandardized B = 0.389; standardized β = 0.683; *p* < 0.001; Figure 6).

**Figure 7:**
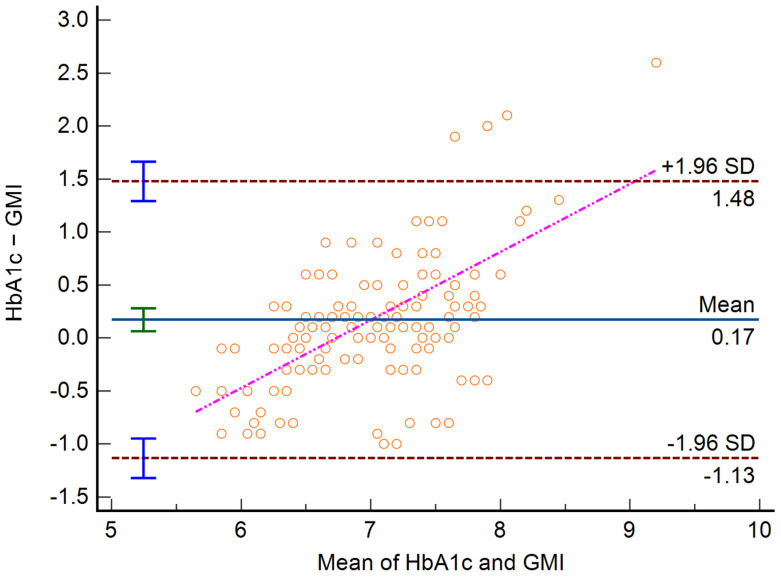
Bland–Altman analysis between HbA1c and GMI.

The Bland–Altman analysis (Figure 7) revealed that usually, in patients with lower HbA1c values, the GMI tends to overestimate this value, while in patients with higher HbA1c values, the GMI tends to underestimate the results. In the regression analysis built with the difference between HbA1c and GMI as the dependent variable and the mean of HbA1c and GMI as the independent variable, a positive slope was observed (y = −4.32 + 0.64x; *p* < 0.001 for both slope and intercept). The arithmetic mean of the Bland–Altman analysis was 0.17 [0.06 to 0.28] 95% CI (*p* = 0.002). The differences between measurements are statistically significant, but their clinical significance is limited, due to the clinically limited relevance of a difference in HbA1c of 0.17 percentage points. However, the importance of the analysis is emphasized by the fact that at lower HbA1c, the GMI overestimates, while in higher HbA1c, the GMI tends to overestimate, leading to a compensation and thus to a low average difference between the measurements.

### Predictors for Differences Between GMI and HbA1c

**Figure 8:**
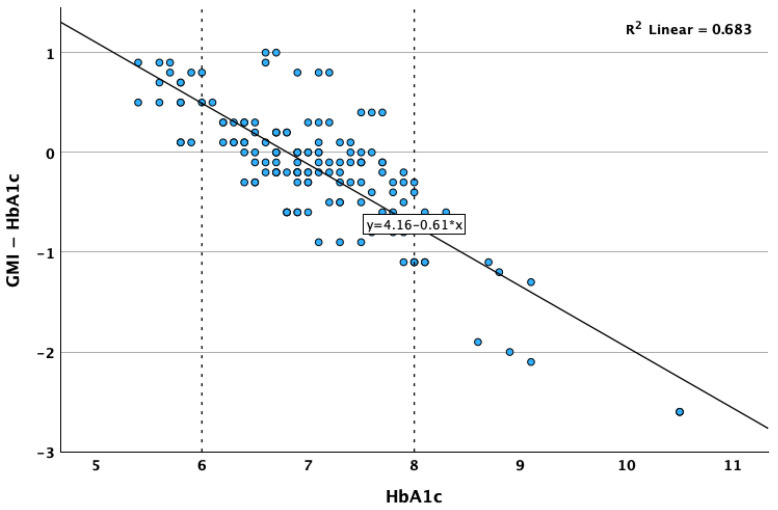
Correlation between HbA1c and difference between GMI and HbA1c.

A reverse, strong and statistically significant correlation was observed between the real value of the HbA1c and the difference between GMI and HbA1c (Spearman’s r = −0.707; *p* < 0.001; Figure 8), indicating that usually, the GMI tends to be higher than the real HbA1c when the HbA1c levels are low, and the GMI tends to be lower than the real HbA1c when the HbA1c levels are high or very high. The magnitude of underestimation by GMI tends to be as high as the real HbA1c value. The GMI performed excellently in the 6–8% HbA1c range, in which all except one of the GMI values differed with less than 1 unit compared to the real HbA1c.

Besides this association, reverse correlations in the difference between GMI and HbA1c were found with the standard deviation of glycemia (Spearman’s r = −0.296; *p* < 0.001) and the coefficient of variation of glycemia (Spearman’s r = −0.299; *p* < 0.001), while positive correlations in the difference between GMI and HbA1c with the time in range were found (Spearman’s r = 0.248; *p* < 0.001). The absolute difference between GMI and HbA1c positively correlated with HbA1c (Spearman’s r = 0.254; *p* < 0.001) and the standard deviation of glycemia (Spearman’s r = 0.189; *p* = 0.022), while it reversely correlated with the time in range (Spearman’s r = −0.190; *p* = 0.021). The correlation matrix between these parameters is presented in Table 1.

## 4. Discussion

The burden of DM extends far beyond the disease itself. It stems not only from the critical need for optimal management of the condition but also from the significant effort required to prevent the onset of various debilitating DM-associated complications [25,26].

Numerous studies have demonstrated a strong association between the quality of glycemic control and the development of diabetes-related complications, particularly microvascular complications (such as chronic kidney disease or diabetic retinopathy), diabetic neuropathy and metabolic dysfunction-associated steatotic liver disease. Patients with poor glycemic management are at a substantially higher risk of developing these complications, which can severely impact their quality of life, as they can impair physical function, cause chronic pain and lead to vision loss as well as to significant healthcare costs [27,28,29,30,31,32,33,34].

Achieving and maintaining tight glycemic control through effective treatment strategies is crucial for mitigating the risk of these complications and optimizing patient outcomes. In T1DM patients, achieving optimal glycemic control is particularly challenging due to the frail balance between the body’s insulin requirements and the exogenous insulin administration through therapy. Since T1DM is an autoimmune disease leading to absolute endogenous deficiency, T1DM patients must carefully monitor their BG levels and precisely adjust their insulin dosages to maintain glycemic homeostasis. This complex management is further complicated by factors such as variations in insulin sensitivity, physical activity, diet and other physiological changes that can disrupt this fragile equilibrium [35,36,37].

The assessment of glycemic control through various metrics is a crucial element in determining whether the glycemic targets set for individuals with T1DM have been successfully achieved.

In the traditional sense, HbA1c levels reflect the weighted average of BG values over the past 2–3 months. American Diabetes Association (ADA) guidelines recommend a targeted value of HbA1c under 7% for non-pregnant patients to be achieved in a safe scenario, acknowledging that individualized HbA1c targets may be appropriate based on factors such as disease duration, age, life expectancy and the presence of cardiovascular disease or other comorbidities. This marker offers valuable insights into an individual long-term glycemic management but it has some limitations since it does not provide data regarding day-to-day GV [38,39,40].

Moreover, obtaining an HbA1c measurement involves several steps, including the need for patients to provide a blood sample, often through a finger prick, followed by laboratory testing. This process can be inconvenient and uncomfortable for patients, especially if frequent monitoring is required. Additionally, the laboratory processing of HbA1c samples necessitates access to healthcare systems and accredited, proficiency-tested facilities, resulting in increased financial costs for both patients and the healthcare system, particularly for individuals with T1DM who require regular monitoring [41,42].

The use of CGMS in clinical practice, along with the need to evaluate glycemic control beyond HbA1c, has led to the development of novel markers derived from CGMS readings that measure the amplitude of GV. In addition, it has allowed the introduction of a standardized metric method for linking HbA1c to CGMS readings and GV metrics. This method was designed to connect the day-to-day glucose fluctuations captured by CGMS with the long-term average glucose levels reflected by HbA1c [43].

The GMI metric was introduced in 2018 as a complementary measure to HbA1c. Derived from the average CGMS reading, the GMI is expressed as a percentage, similar to HbA1c. The GMI offers several advantages, including improved patient engagement. Since the GMI can be calculated at home using CGMS data, individuals need to actively track their glycemic control between HbA1c lab tests or medical appointments. The GMI can also provide a more comprehensive assessment of glycemic control by accounting for both short-term and long-term glucose fluctuations. The metric can be configured to analyze data from the past 10 to 90 days, enabling clinicians and patients to gain insights into glycemic patterns over different time frames. This, in turn, can empower patients to make more informed decisions about their diabetes management [44,45].

Furthermore, the use of the GMI can have a positive impact in the context of diabetes burnout, as it reduces the need for blood draws while providing real-time feedback on glycemic control without requiring additional effort from the patient. However, the GMI should be used with caution in this situation. It is essential to set individualized, realistic and safe goals in such instances/cases, since an excessive focus on constantly achieving the ideal GMI value may increase pressure for the patient, potentially exacerbating the emotional stress [17,46].

Another key advantage of using the GMI is that it can be superior to HbA1c in certain specific clinical situations.

Since HbA1c represents the amount of glucose that binds to hemoglobin, its value can be influenced by various diseases and conditions that affect the lifespan of red blood cells (RBC), leading to falsely increased or reduced values [42].

Hemoglobinopathies, such as sickle cell disease, which involve an abnormal hemoglobin variant may result in a lower HbA1c values. This occurs because the abnormal hemoglobin structure can affect the glycation process, leading to a reduced HbA1c level [47].

Iron deficiency anemia, the most common form of anemia, can cause an alteration in hemoglobin structure, making it more prone to glycation, leading to higher HbA1c values. Conversely, hemolytic anemia, which shortens the lifespan of erythrocytes, may lead to lower HbA1c values [48,49].

Furthermore, other non-hematologic pathologies, such as chronic kidney disease (CKD) and liver disease, can also interfere with HbA1c measurements. These conditions can impact the turnover of RBC, leading to inaccurate HbA1c test results [50,51].

A study involving 641 patients compared HbA1c values with GMI values in patients with various comorbidities. The results showed a statistically significant discordance between HbA1c and GMI in patients with a Glomerular Filtration Rate below 60 mL/min/1.73 m^2^. This discrepancy is likely due to the impact of CKD on RBC turnover, which can affect the accuracy of HbA1c as a marker of glycemic control in this population [52].

Nevertheless, the accuracy of the GMI could interfere with several factors. Firstly, since the GMI is exclusively based on CGMS readings, any inaccuracies in the CGMS device itself or issues with the calibration process can lead to an inaccurate GMI value. This is a key limitation, as the reliability of the GMI is contingent on the performance of the underlying CGMS technology. Secondly, long-term GMI measurement is strongly influenced by the user’s adherence to wearing the glycemic sensor. The typical lifespan of a glycemic sensor is 7–14 days, which means that for a 90-day GMI calculation, the user would need to wear and change multiple sensors. Furthermore, each sensor changes results in a downtime of at least 2 h during which no glucose readings can be obtained, as the transmitter needs to be recharged, and the new sensor requires a warm-up period. At the same time, discrepancies between the GMI and HbA1c may occur due to factors that are influencing the HbA1c, like anemia, hemoglobinopathies or variable glycation rates. This intermittent data collection can introduce gaps and variability in the CGMS data used to calculate the GMI, potentially impacting its accuracy over longer time frames [17,53].

The GMI value also depends on the Mean Absolute Relative Difference (MARD) of the CGMS. Since MARD reflects the accuracy of the CGMS readings, glycemic sensors with higher MARD, translating as a lower accuracy of BG readings, provide an unreliable GMI measure [54].

Even so, validating the GMI as an indicator in current medical practice has become imperative to provide complementary information alongside HbA1c. This would enable a more comprehensive assessment of glycemic control, by incorporating both long-term and short-term measures. When integrated alongside existing metrics, this new measure has the potential to enhance the understanding of a patient’s BG management.

The key strengths of this study include a large cohort of participants, with the sample size calculated to ensure statistical power. All patients were recruited from two separate medical centers, which enhances the generalizability of the findings. The study population included individuals receiving both basal/bolus insulin therapy and continuous subcutaneous insulin infusion, allowing for a comprehensive evaluation of glycemic variability across different treatment modalities. To our knowledge, this is the first study of its kind conducted in the Romanian patient population, providing important insights into glycemic control in this specific context. All participants used the same type of CGMS device, minimizing potential variability introduced by different measurement technologies.

The weaknesses of this study are represented by the non-continuous use of the CGM system, as this is a common issue encountered in real-world clinical practice. However, patients who wore the device for less than 80% of the study duration were excluded.

Similar to this research, a study conducted in China that included 91 adults previously diagnosed with T1DM, across five hospitals, revealed a statistically significant linear relationship between GMI and HbA1c values. However, in patients exhibiting high GV and a high blood glucose index, discrepancies were observed between these two parameters. This suggests that a combined use of GMI and HbA1c may be necessary to provide a more comprehensive assessment of glycemic control in T1DM patients, particularly in those with increased GV and heightened blood glucose excursions [55].

Other research regarding GMI and laboratory-measured HbA1c showed various discrepancies yet suggested that the difference between these two remains relatively stable for each patient over time. These differences are highlighted in a study that analyzed data from multiple CGMS records. The results showed that up to 20% of patients had nearly the same values of HbA1c and GMI; however, the discrepancies increased as the mean blood glucose rose [56].

Lastly, considering that anthropometric variables such as gender and age can influence average glycemic levels through various mechanisms, it is important to further develop the GMI calculation formulas. Erythrocyte lifespan and blood count (men typically have a higher erythrocyte count and a slightly longer lifespan of red blood cells compared to women), hormonal differences (the menstrual cycle and hormonal levels can impact insulin sensitivity), insulin resistance (which tends to increase with age as insulin levels decrease) and oxidative stress (aging is associated with increased oxidative stress, which can damage red blood cells and shorten their lifespan) all contribute to variations in glycemic levels and, in turn, impact the GMI values. By incorporating models that account for these influential parameters, the GMI calculation could be refined to provide more accurate and personalized insights into glycemic control [57,58,59].

These characteristics likely reflect each individual’s unique glycemic profile that influences the relationship between average glucose levels and hemoglobin glycation. Further larger, muti-centric and multi-national studies examining the GMI and HbA1c in relation to physiological and pathophysiological factors, such as age or associated comorbidities, are needed to provide a more comprehensive understanding of the GMI and its clinical implications.

## 5. Conclusions

The GMI is a reliable, immediate, point-of-care tool that provides a valuable assessment of overall glycemic control in patients with T1DM when using a CGMS. The differences between GMI and HbA1c measurements are not clinically significant, indicating a strong concordance between these two methods. The GMI has demonstrated good concordance, correlation and internal consistency with HbA1c measurements in patients with T1DM. However, it is important to note that the relationship between GMI and HbA1c is not always straightforward. In patients with low HbA1c, the GMI tends to overestimate the average glycemia, while in patients with high HbA1c, the GMI tends to underestimate the glycemia. This discrepancy highlights the need for healthcare providers to be aware of the potential limitations of the GMI and to interpret the results in the context of the individual patient’s clinical presentation and other relevant factors.

## Figures and Tables

**Table 1:** Correlation matrix.

		GMI-HbA1c	Absolute difference (GMI-HbA1c)	HbA1c	Standard Deviation of Glycemia	Time In Range
Percentage Error (GMI vs. HbA1c)	Correlation Coefficient	−0.205				
	*p*-value	0.013				
Absolute difference (GMI-HbA1c)	Correlation Coefficient	−0.295	--			
	*p*-value	<0.001	.			
HbA1c	Correlation Coefficient	−0.707	0.254	--		
	*p*-value	<0.001	0.002	.		
Standard Deviation of Glycemia	Correlation Coefficient	−0.296	0.189	0.656	--	
	*p*-value	<0.001	0.022	<0.001	.	
Time In Range	Correlation Coefficient	0.248	−0.190	−0.637	−0.794	--
	*p*-value	0.002	0.021	0.000	0.000	.
Coefficient of variation	Correlation Coefficient	−0.299	0.161	0.349	0.801	−0.513
	*p*-value	<0.001	0.051	<0.001	<0.001	<0.001

## Data Availability

The data presented in this study are available on request from the corresponding author due to restrictions imposed by the General Data Protection Regulation procedure in the hospitals in which this study was conducted.
